# Copper in colorectal cancer patients: a systematic review and meta-analysis

**DOI:** 10.1093/carcin/bgaf001

**Published:** 2025-01-23

**Authors:** Carlos Muñoz-Bravo, Inés Marín-Burdallo, Lucas González-Herrera, Carla González-Palacios Torres, Macarena Lozano-Lorca, José Juan Jiménez-Moleón, Rocío Olmedo-Requena

**Affiliations:** Department of Public Health and Psychiatry, School of Medicine, University of Málaga, 29071 Málaga, Spain; Biomedical Research Institute of Malaga (IBIMA), 29010 Málaga, Spain; Departamento de Medicina Preventiva y Salud Pública, Universidad de Granada, 18016 Granada, Spain; Department of Forensic Medicine, Faculty of Medicine, University of Granada, Avenida de la Investigación 11, 18016 Granada, Spain; Departamento de Medicina Preventiva y Salud Pública, Universidad de Granada, 18016 Granada, Spain; Departamento de Medicina Preventiva y Salud Pública, Universidad de Granada, 18016 Granada, Spain; Instituto de Investigación Biosanitaria ibs.GRANADA, 18014 Granada, Spain; Centro de Investigación Biomédica en Red de Epidemiología y Salud Pública, Instituto de Salud Carlos III, 28029 Madrid, Spain; Departamento de Medicina Preventiva y Salud Pública, Universidad de Granada, 18016 Granada, Spain; Instituto de Investigación Biosanitaria ibs.GRANADA, 18014 Granada, Spain; Centro de Investigación Biomédica en Red de Epidemiología y Salud Pública, Instituto de Salud Carlos III, 28029 Madrid, Spain; Departamento de Medicina Preventiva y Salud Pública, Universidad de Granada, 18016 Granada, Spain; Instituto de Investigación Biosanitaria ibs.GRANADA, 18014 Granada, Spain; Centro de Investigación Biomédica en Red de Epidemiología y Salud Pública, Instituto de Salud Carlos III, 28029 Madrid, Spain

**Keywords:** copper, copper/zinc ratio, colorectal cancer, cancer, meta-analysis

## Abstract

Several clinical studies have evaluated the relationship between copper on colorectal cancer (CRC), but the results are contradictory. This study aimed to conduct a systematic review and meta-analysis to investigate copper measured in two biological matrices (serum/plasma/blood and tissue) and dietary intake in CRC patients compared to healthy controls. We conducted a comprehensive and systematic search in PubMed, Scopus, Embase, and Web of Science. We included studies that reported copper levels in serum/plasma/blood, tissue, or from the diet, with an observational study design (cohort and case–control studies). Study quality was assessed with the Newcastle–Ottawa scale and potential causes of heterogeneity were evaluated. Standardized mean differences (SMD) with 95% confidence interval (CI) were pooled using random-effect models. Overall pooled odds ratio and 95% CI for the risk of CRC were calculated. Twenty-six studies (23 case–control and 3 cohort studies) with a total of 227 354 participants were included. Most of the studies presented low (50%) or moderate quality (42.3%). No differences in serum/plasma/blood copper levels (SMD = 0.23; 95% CI: −0.23, 0.70; *I*^2^ = 97.3%, 19 studies), tissue copper levels (SMD = −1.69; 95% CI: −3.41, 0.03; *I*^2^ = 85.6%, 2 studies), or copper/zinc ratio (SMD = 1.19; 95% CI: 0.54, 1.84; *I*^2^ = 95.3%, 6 studies) were found between CRC patients and healthy controls. Regarding dietary copper, CRC patients had a lower intake (SMD = −0.27; 95% CI: −0.51, −0.03; *I*^2^ = 0.0%, 2 studies). No differences were found in copper levels between CRC patients and healthy controls. However, evidence shows mostly low or moderate quality, and results were heterogeneous. More prospective studies with an adequate methodological approach are needed.

## Graphical Abstract

**Graphical Abstract:**
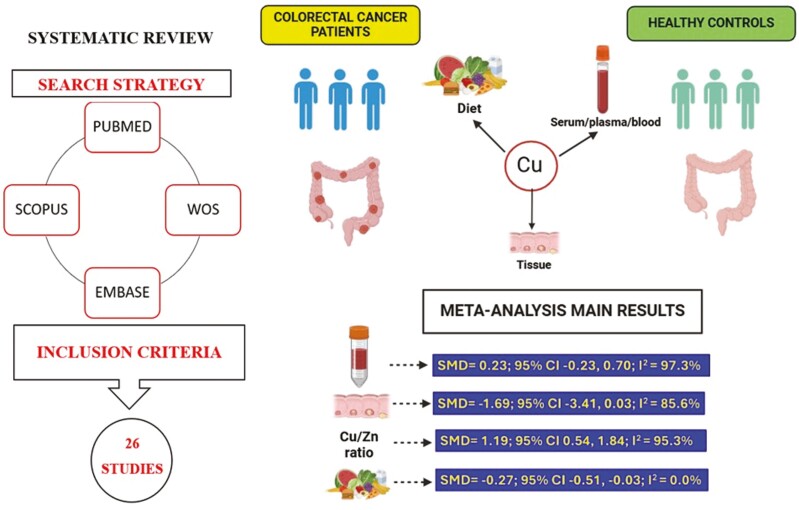

## Introduction

Cancer, together with cardiovascular disease, represents the greatest public health problem today [1,2]. In particular, colorectal cancer (CRC) is the third most common cancer (around 2 million new cases diagnosed in 2020) and the second in mortality (approximately 1 million deaths in 2020) worldwide. It is estimated that between 2020 and 2040, both the number of new cases and the number of deaths will increase, respectively [3,4].

Age is the most important nonmodifiable risk factor for CRC. The majority of new cases occur after the age of 50–55 years, with a marked increase in incidence in men compared to women [4]. Other risk factors, such as obesity, sedentary lifestyle, smoking, diabetes, high consumption of red and processed meats, as well as *Helicobacter pylori* infection, have also been associated with CRC [5–7].

In adults, normal serum levels range between 73 and 206 µg/dL according to Mayo Clinic laboratories, whereas in normal colorectal tissue, some studies have described copper concentrations between 1037 and 1790 µg/kg [8–10]. Copper is an essential element for the organism that forms part of proteins with important biological functions. However, it may also promote the formation of reactive oxygen species, leading to the oxidation of organic biomolecules [11]. Copper homeostasis is crucial, as dysregulation of copper metabolism can increase copper levels. Higher copper levels have been associated with an increased risk of cardiovascular disease [12], neurodegenerative disease [13], as well as lung and breast cancer [14,15]. This may be due to a process called cuproplasia [16]. The increased metabolic activity of the tumour cells demands higher requirements from these cells, which could explain the increased level of copper in the pathologies mentioned above.

Maintaining correct trace elements homeostasis is essential for the body to function. Human health can be adversely affected by excess or deficiency of trace elements as a consequence of dyshomeostasis. It has been shown that tumour cells have increased copper levels compared to normal cells. This increase is known as cuproplasia. At the same time, this increase in intracellular copper levels causes a type of programmed cell death called cuproptosis, which is closely associated with some diseases, particularly cancer. Copper chelators and copper ionophores are shown as strategies to increase or decrease the intracellular concentration of copper ions and to promote or suppress cuproptosis [17,18].

Diet is the main source of copper intake. Dietary copper mainly comes from organ meats (especially liver), oysters, whole grains, and nuts. Diets with a high consumption of red meat have been associated with an increased risk of CRC. This could be due to the high copper content of this type of meat. Thus, Western diets are probably to provide a higher amount of copper than Eastern diets [19].

To date, several studies have examined the relationship between copper and CRC. However, the results obtained are contradictory. For instance, while some investigations reported higher serum copper levels in CRC patients compared to healthy controls [20,21], others found no difference in copper levels between CRC patients and controls [22,23]. Likewise, some studies quantified higher copper concentrations in controls versus CRC patients [24,25]. A meta-analysis comparing serum/plasma/blood copper levels between CRC patients and controls has recently been published [26]. However, there are some aspects to consider: (i) the inclusion of case–control studies with prevalent cases, which do not allow causality to be verified; (ii) the need for a more in-depth analysis of heterogeneity, considering potential sources of relevance, such as the period of publication or the quality of the studies; and (iii) the absence of certain studies that analyse the association between copper and CRC cancer, which is far from a systematic review that should include all the studies published on the subject.

Furthermore, to our knowledge, no meta-analysis has been published considering tissue copper concentration, dietary copper intake, or copper/zinc ratio between CRC patients and healthy subjects, nor has the association between serum copper levels and CRC risk been previously examined; so, the present work would be the first meta-analysis to address these issues. Thus, we conducted a systematic review and meta-analysis aiming to evaluate the current epidemiologic evidence on (i) copper levels in serum/plasma/blood, tissue, and through the diet of CRC patients and healthy subjects; (ii) serum copper/zinc ratio in CRC patients and healthy subjects. To answer this objective, the methodological quality of the studies and possible sources of heterogeneity between them were analysed.

## Methods

This systematic review and meta-analysis were conducted according to the 2020 update of the Preferred Reporting Items for Systematic Review and Meta-Analysis (PRISMA) statement [27]. The protocol was previously registered in PROSPERO (CRD42023480709). Ethical approval for this study was not required as the data employed did not reveal patient identifiers.

The focused question was based on Participants/Population, Exposure, Comparator, and Outcome (PECO) strategy: (P) men and women older than 18 years old (no upper limit); (E) copper measured in two biological matrices (serum/plasma/blood and tissue) and dietary intake; (C) participants without exposure or with low copper exposure; (O) CRC (Supplementary Table 1).

### Data sources and search strategy

We performed online literature searches through electronic platforms PubMed, Scopus, and Embase from the inception until 7 November 2023. The search was kept active from 8 November 2023 to 12 November 2024, using the alert systems of each electronic platform. An additional search was carried out through Web of Science from the inception until 12 November 2024. We used search terms related to ‘copper’, ‘trace elements’, ‘mineral intake’, ‘colon cancer’, ‘colorectal cancer’, and ‘colorectal tumor’. The search strategies are available in Supplementary Table 2. Besides, a manual search by browsing the references of all the selected articles to ensure that eligible studies were not lost.

### Study selection

A study was considered eligible if (i) it determines the levels of copper in serum/plasma/blood, tissue, or diet intake; (ii) the outcome of interest was CRC; (iii) it was a cohort, case–cohort, or hybrid study; (iv) it was written in English or Spanish; and (v) with full-text available. Nonhuman studies, literature or systematic reviews, meta-analyses, umbrella reviews, experimental studies, trial protocols, letters to the editor, editorials, book chapters, abstracts, or theses were not considered.

Two investigators (C.M.-B. and I.M.-B.) independently conducted a search and identified the eligible articles. After removing duplicates, a first screening by title and abstract was done. Articles that met inclusion criteria were assessed by full-text reading. Disagreements were resolved through discussion with the assistance of two researchers (M.L.-L. and J.J.J.-M.).

### Data extraction

The following data were extracted by two independent researchers (C.M.-B. and I.M.-B.), using a pre-designed form, for each eligible study: (1) first author (year); (2) country; (3) sample size; (4) age of participants; (5) sex (% female); (6) copper levels; (7) copper/zinc ratio; (8) effect estimates and 95% confidence intervals (CIs); (9) source of copper (serum/plasma/blood, tissue or diet); (10) method for determination; (11) Newcastle–Ottawa Scale (NOS) score; and (12) adjustment variables in multivariable analysis. In addition, for cohort studies, the following were collected: (13) number of incident cases and (14) follow-up (years). Discrepancies were resolved through face-to-face discussions and checked by two researchers (M.L.-L. and J.J.J.-M.).

### Quality assessment

The NOS [28] was used to evaluate the quality of included studies. Two researchers assessed quality independently (C.M.-B. and I.M.-B.). This scale uses a star system to assess the study quality based on three aspects: (1) selection of study groups; (2) comparability of study groups; and (3) determination of the exposure or outcome of interest for case–control or cohort studies. Each study can be rated with a maximum of nine stars. Since there is no specific reference for categorization, the included studies were classified into high (8–9 stars), moderate (6–7), or (3) low (≤5) quality. Consensus was reached through discussion and consultation of two additional researchers (M.L.-L. and J.J.J.-M.) when needed.

### Statistical analysis

To examine the relationship between copper and CRC, we conducted a meta-analysis. First, we analysed the mean differences in copper levels from different sources (serum/plasma/blood, tissue, and diet) between CRC patients and the healthy group. Second, for those studies that provided data, mean differences in the copper/zinc ratio were studied in CRC patients and the control group; and third, we studied the association between copper and the risk of CRC. To calculate the difference of means, sample size, and mean (standard deviation [SD]), copper levels were extracted from each included study for both CRC cases and healthy controls. If medians and interquartile range were reported, means and SDs were estimated using the equation of Wan X *et al*. [29]. If means and standard errors (SEs) were reported, SDs were estimated through the following equation: SD = SE × $\sqrt{n}$. Whereas if means and 95% CIs were reported, SDs were estimated by the following equation: SD = √ *n* × (upper limit − lower limit)/4.128 (for fewer than 60 participants in each group) [30]. Due to several studies that reported copper levels in different units, Hedges’s *g* standardized mean differences (SMDs) with their 95% CIs were used to calculate the effect size.

For studies that analysed the association between copper and the risk of CRC, the measure of association (odds ratio [OR] or hazard ratio [HR]) and 95% CIs were extracted from the full-adjusted model. For this, copper levels were used as a continuous and categorical variable. For categorical variables, quantile 1 was used as the reference group. To obtain the effect estimate, we use the overall pooled OR.

The meta-analyses were conducted using a random-effects model. The heterogeneity between studies was evaluated using the Cochrane’s *Q* and *I*^2^ tests. Heterogeneity was considered as low for values between 25% and 50%, moderate for 50%–75%, and high for values higher than 75% [31]. Cumulative meta-analyses were conducted by date of publication, adding a more recent study each time to assess the stability of the pooled effect estimated. To investigate potential sources of heterogeneity, subgroup analyses, and meta-regression were performed by continent, country, copper determination method, publication year, NOS score, and sample size. Therefore, we conducted a sensitivity analysis, omitting one study each time to assess whether the estimated effect differed significantly when each study was excluded from the meta-analyses. The presence of publication bias was assessed visually using funnel plots. The Egger’s test was used for formal statistical evaluation of funnel plot asymmetry. The trim and fill method was also used to adjust for funnel plot asymmetry. All statistical analyses were conducted using Stata SE v.18.0 (Stata Corp, College Station, TX). Statistical tests were two-sided, with statistical significance evaluated at *P-*values <.05.

## Results

### Search results

**Figure 1:**
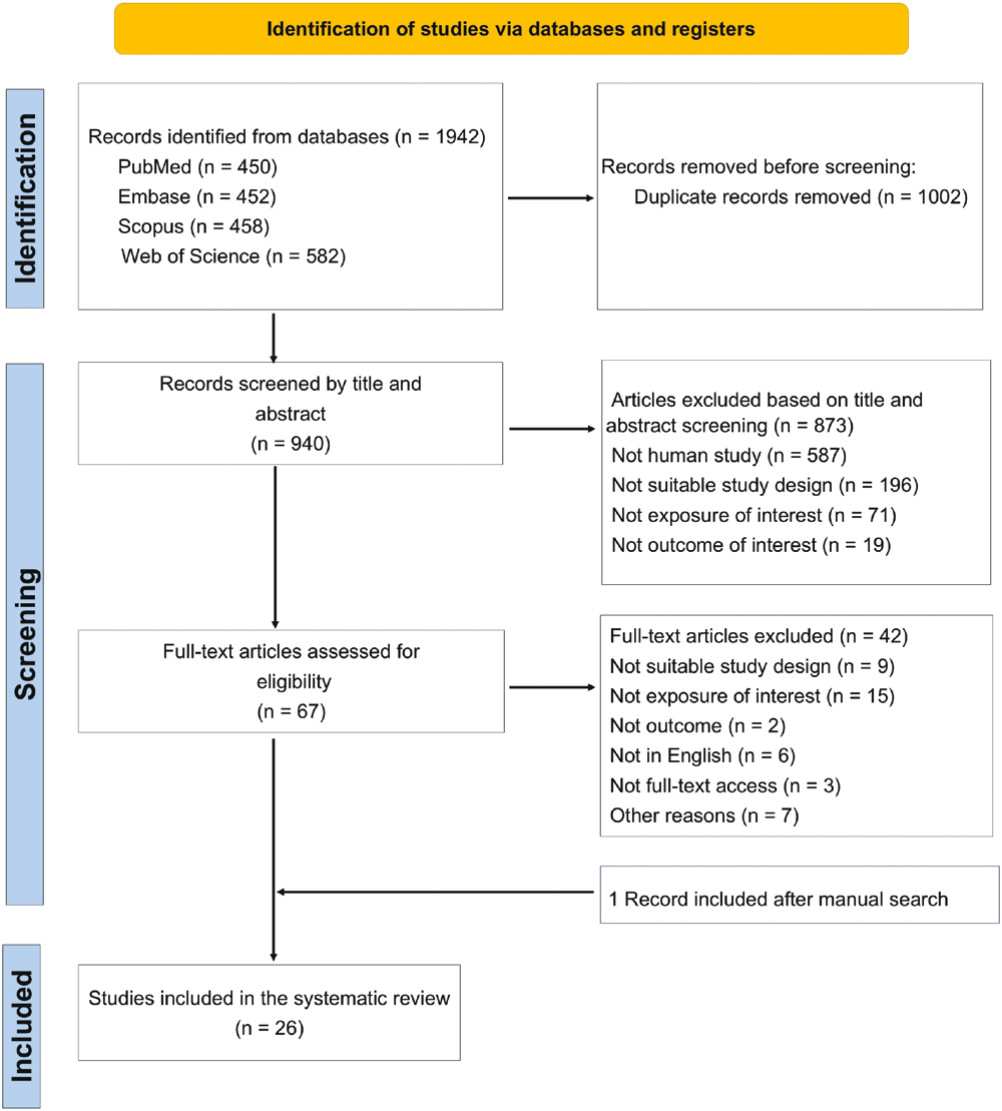
Flowchart of study selection

Figure 1 shows the results of the literature search and study selection. A total of 1942 records were initially identified, 450 from PubMed, 452 from Embase, 458 from Scopus, and 582 from Web of Science. After excluding 1002 duplicate records and screening the remaining 940 articles by title and abstract, we excluded 873 as they were irrelevant for our objective. After a full-text evaluation of 67 articles, 42 were finally excluded (Supplementary Table 3). Therefore, 25 articles were identified in the first phase. In addition, an article was added from a manual literature search. Finally, a total of 26 articles were included in this systematic review and meta-analysis [8,10,20–25,32–49].

### Characteristics of the studies

Study characteristics are shown in Tables 1 and 2 for case–control and cohort studies, respectively. A total of 227 354 participants were included in this systematic review. The number of participants in the studies ranged from 21 to 118 210, and the age ranged from 30 to 94 years. The articles were published between 1988 and 2024. Fourteen out of 26 studies included (53.8%) measured copper in serum [8,20,21,23–25,32–34,36,41,43,45,48], 2 in plasma (7.7%) [22,44], 3 in blood (11.5%) [35,38,39], 1 in tissue (3.8%) [10], 1 in plasma and dietary intake (3.8%) [40], 1 in serum and tissue (3.8%) [46], and 4 from diet (15.4%) [37,42,47,49].

**Table 1.** Characteristics of included case–control studies.

		Colorectal patients					Healthy controls									
First author (year)	Country	*n*	Age (year) [mean (SD) or range]	Female (%)	Copper levels [mean (SD)]	Cu/Zn ratio [mean (SD)]	*n*	Age (year) [mean (SD) or range]	Female (%)	Copper levels [mean (SD)]	Cu/Zn ratio [mean (SD)]	Effect estimates (95% CI)	Adjustment variables	Source	Method for determination	NOS score
Li, 2023 [21]	China	101	60.77 (10.28)	46.53	1003.77 (279.29) μg/L	NR	60	45.13 (16.76)	73.33	815.56 (137.92) μg/L	NR	OR_per1-unit increment_: 1.01 (1.00–1.01)	Age, drinking, gender, and smoking	Serum	ICP-MS	7
Saxena, 2023 [32]	India	52	45.5	44.2	180.14 (104.12) μg/dL	NR	52	NR	NR	90.83 (7.79) μg/dL	NR	NR		Serum	Colorimetric	7
Mahmood, 2022 [24]	Pakistan	165	42.85 (13.02)	48	1.114 (0.490) μg/g	NR	151	41.93 (11.01)	54	2.033 (0.876) μg/g	NR	NR		Serum	FAAS	4
Mardan, 2022 (Female) [33]	Iraq	24	47.7 (15.9)	100	176.8 (240) ppb	1.35 (0.30) ppb	15	47.3 (7.3)	100	152.5 (67.01) ppb	0.10 (0.04) ppb	NR		Serum	FAAS	4
Türkdogan, 2022 [34]	Turkey	24	NR	NR	2.870 (0.06) μg/mL	NR	30	NR	NR	2.334 (0.08) μg/mL	NR	NR		Serum	FAAS	5
Baszuk, 2021 [35]	Poland	187	32-94	44	1031 (657–2043)^a^ μg/L	NR	187	31–91	44	864 (589–1433)^a^ μg/L	NR	OR_Q4vsQ1_: 12.7 (4.98–32.3)	Matched for sex, smoking and year of birth	blood	ICP-MS	7
Nozadi, 2021 [10]	Iran	24	64.71 (12.78)	47.6	908.35 (145.7) μg/kg	NR	17	51.65 (19.41)	50.8	1036.9 (141.9) μg/kg	NR	NR		Tissue	ICP-MS	7
Al-Ansari, 2020 [25]	Iraq	30	50.75 (11.93)	NR	30.38 (2.57) μg/dL	NR	30	49.97 (10.85)	NR	80.11 (3.21) μg/dL	NR	NR		Serum	FAAS	5
Natajomrani RA, 2020 [36]	Iran	40	60 (9.42)	50	83.9 (15.61) μg/dL	NR	40	43 (13.4)	80	101.4 (15.69) μg/dL	NR	NR		Serum	Spectrophotometric	6
Nawi, 2020 [37]	Malaysia	102	63.16 (10.33)	38.2	1.54 (0.52) mg	NR	102	60.37 (10.70)	52.9	1.69 (0.56) mg	NR	NR		Diet	FFQ	5
Ranjbary, 2020 [38]	Iran	40	55.87 (11.9)	37.5	0.82 (0.49) μg/mL	NR	40	55.87 (11.9)	37.5	0.61 (0.32) μg/mL	NR	NR		blood	ICP-OES	5
Wang, 2020 [39]	China	93	58.6 (11.7)	53.8	1.08 (0.22) μg/L	NR	48	NR	NR	0.93 (0.11) μg/L	NR	NR		blood	ICP-MS	3
Stepien, 2017 [20]	Multi-European	966	58.6 (7.1)	NR	138.6 (30.5) μg/dL	1.49 (0.43) μg/dL	966	58.6 (7.1)	NR	135.8 (30.8) μg/dL	1.45 (0.39) μg/dL	OR_Q5vsQ1_: 1.50 (1.06–2.13)	Matching factors plus alcohol, BMI, education, fruit, red and processed meat intake, smoking, sex-specific physical activity, and vegetable	Serum	TXRF	9
Figueiredo-Ribeiro, 2016 [40]	Brazil	46	68.5 (12.7)	52.2	120 (19.7) μg/dL	1.59 (0.38) μg/dL	28	61.4 (8.6)	50	106 (20.5) μg/dL	1.35 (0.30) μg/dL	NR		Plasma	FAAS	5
Figueiredo-Ribeiro, 2016 [40]	Brazil	46	68.5 (12.7)	52.2	1.24 (0.71) mg	NR	28	61.4 (8.6)	50	1.42 (0.66) mg	NR	NR		Diet	SQFFQ	5
Khosdel, 2016 [23]	Iran	119	55.84 (14.5)	46.2	137.5 (122.38) μg/dL	2.95 (3.95) μg/dL	128	52.43 (7.77)	50.8	160.68 (45.12) μg/dL	1.31 (0.75) μg/dL	NR		Serum	FAAS	5
Kucukhuseyin, 2015 [22]	Turkey	80	62.94 (12.27)	41.3	144.4 (6.91) μg/dL	NR	115	NR	NR	158.9 (5.70) μg/dL	NR	NR		Plasma	AAS	6
Al Faris, 2011 [41]	Saudi Arabia	256	48.53 (16.34)	31.25	154.60 (91.71) μg/dL	NR	180	47.01 (17.78)	31.1	152.08 (112.56) μg/dL	NR	NR		Serum	Colorimetric	7
Senesse, 2004 [42]	France	171	30-79	36.3		NR	309	30-79	48.5		NR	OR_Q4vsQ1_: 2.40 (1.28-4.51)	Age, BMI, energy intake, physical activity and sex	Diet	DHQ	7
Magálová, 1999 [43]	Slovak Republic	40	38-76	25	18.23 (3.30) μmol/L (colon cancer)	NR	74	44-72	54.1	17.60 (3.07) μmol/L	NR	NR		Serum	FAAS	5
Stefanati, 1995 [44]	Italy	31	58.9 (8.6)	51.6	1244.5 (260) μg/L	NR	51	54.4 (6.3)	56.9	974 (192.82) μg/L	NR	NR		Plasma	ICP-MS	6
En-Ling, 1993 [45]	China	23	51 (14.4)	26.1	17.9 (4.8) μmol/L	1.17 (0.29) μmol/l	35	38 (11.84)	42.9	15.9 (1.78) μmol/L	1.10 (0.18) μmol/l	NR		Serum	I-EC	4
Gupta, 1993 [8]	India	30	40 (10)	13.3	166.0 (33.9) μg/dL	1.86 (0.65) μg/dl	30	46 (9)	NR	98.8 (24.3) μg/dL	0.86 (0.15) μg/dl	NR		Serum	AAS	5
Martín-Mateo, 1988 [46]	Spain	10	NR	NR	61.10 (39.37) μmol/L	NR	10	NR	NR	17.16 (28.35) μmol/L	NR	NR		Serum	AAS	4
Martín-Mateo, 1988 [46]	Spain	11	NR	NR	0.97 (0.37) μg/g	NR	10	NR	NR	9.5 (4.5) μg/g	NR	NR		Tissue	AAS	4

Abbreviations: AAS, atomic absorption spectrometry; DHQ, Diet History Questionnaire; FAAS, flame atomic absorption spectrometry; FFQ, Food Frequency Questionnaire; ICP-MS, inductively coupled plasma mass spectrometry; ICP-OES, inductively coupled plasma optical emission spectrometry; I-EC, ion-exchange chromatography; NOS, Newcastle–Ottawa Scale; NR, not reported; OR, odds ratio; SQFFQ, semi-quantitative food frequency questionnaire; TXRF, total reflection X-ray fluorescence.

^a^Mean (range).

**Table 2.** Characteristics of included cohort studies.

First author (year)	Country	*n*	Nº of incident cases	Female (%)	Age (year) [mean (SD)]	Follow-up (years)	Source	Methods for determination	Effect estimates (95% CI)	Adjustment variables	NOS score
Li, 2024 [49]	USA	101 686	1100	51.37	62.40 (5.28)	Median: 11.3	Diet	DHQ	HR_Q4vsQ1_: 0.80 (0.68–0.95)	Age, body mass index, education, drinking status, family history of colorectal cancer, family history of any cancer, marital status, race, randomization arm, sex, smoking status	6
Jin, 2023 [47]	UK	118 210	1466	55.4	55.87 (7.83)	Mean: 12.8	Diet	Web-based, self-administered 24-h dietary questionnaire	HR_per 1-sd increment_: 0.98 (0.91–1.05)	Age at recruitment, body mass index, bowel screening, diabetes, education, ethnicity, family history of CRC, physical activity, regular aspirin use, sex, smoking, total energy intake, Townsend deprivation index	8
Cabral, 2021 [48]	Germany	2106	219	Not reported for colorectal outcome	Not reported for colorectal outcome	Median:10.7 Interquartile range: 1.6	Serum	ICP-MS/MS	HR_per 1-sd increment_: 1.29 (1.05–1.59)	Age, alcohol intake, anti-hypertensive medication, body mass index, educational attainment, Fe, I, lipid-lowering medication, Mediterranean score, Mn, physical activity, prevalent hypertension, Se, sex, smoking status, vitamin and mineral preparations, waist circumference, Zn.	7

Abbreviations: DHQ, Diet History Questionnaire; HR, hazard ratio; ICP-MS, inductively coupled plasma-tandem mass spectrometry; NOS, Newcastle–Ottawa Scale.

More than half of the studies were conducted in Asian countries (*n* = 14; 53.8%) [8,10,21,23–25,32,33,36–39,41,45], while eight studies (30.8%) were conducted in Europe [20,35,42–44,46–48]. American countries [40,49] and Eurasia (Turkey) [22,34] were also represented with two studies, respectively. Regarding the epidemiological study design of the studies, 23 (88.5%) were case–control studies [8,10,20–25,32–46], and 3 (11.5%) were cohort studies [47,48]. In relation to study quality, 13 studies (50%) were assessed as low, 11 (42.3%) as moderate, and 2 (7.7%) as high quality (Supplementary Table 4).

### Main results for the association between copper and colorectal cancer

#### Copper

##### Serum/plasma/blood

**Figure 2:**
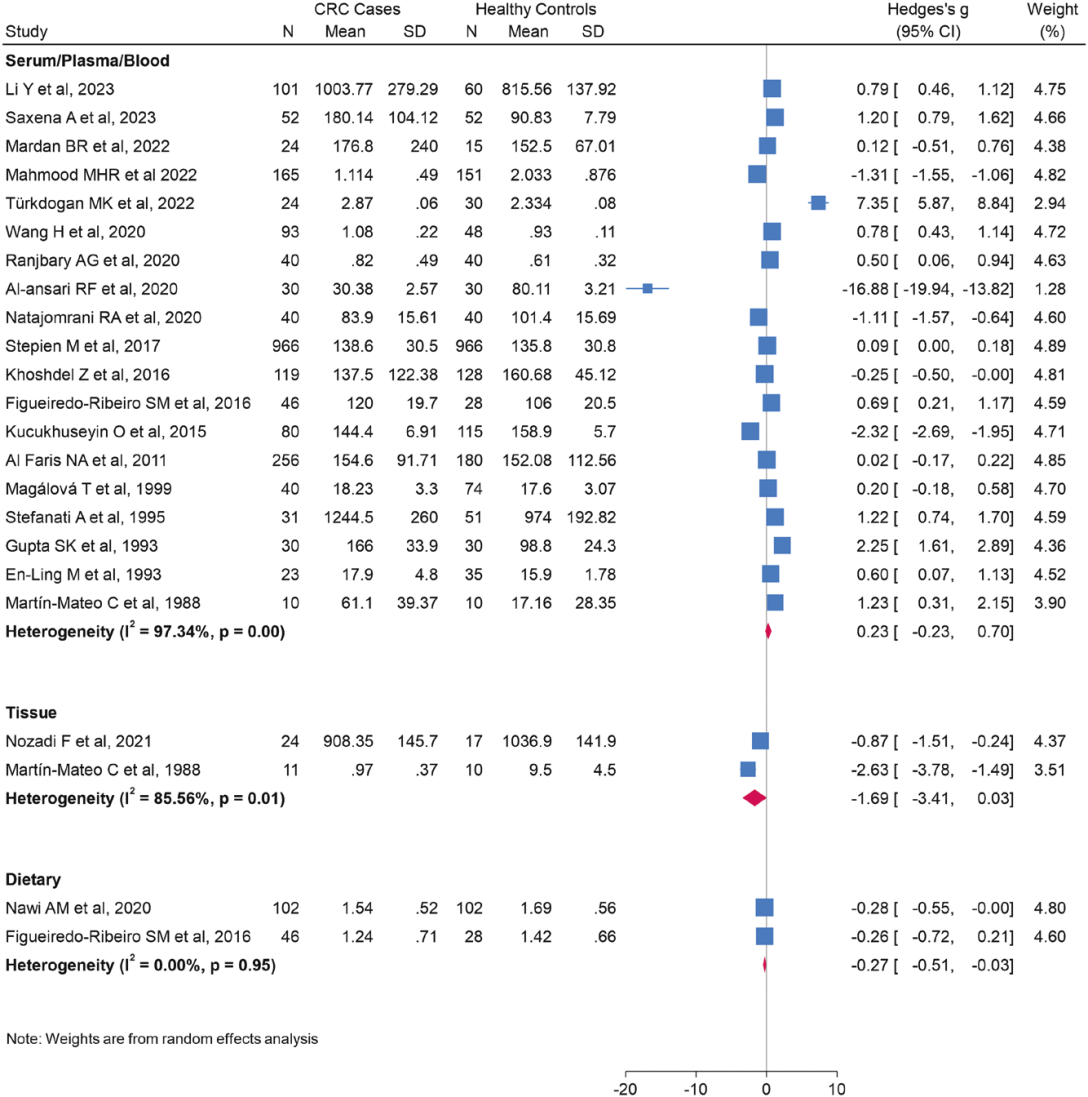
Forest plot of Hedges’s *g* SMD for serum/plasma/blood and tissue copper levels and dietary copper intake

The pooled mean difference of 19 studies included in the meta-analysis showed that there were no differences in serum/plasma/blood copper levels in CRC patients and controls (SMD = 0.23; 95% CI: −0.23, 0.70), observing a very high heterogeneity (*I*^2^ = 97.34%; *P* = .00) (Figure 2). When performing the subgroups analysis (Table 3), heterogeneity remained high for all of them except for inductively coupled plasma mass spectrometry (ICP-MS) methods (*I*^2^ = 17.72%; *P* = .30) and studies conducted in China (*I*^2^ = 0.0%; *P* = .81). Both in the analysis for the subgroup of studies using the ICP-MS method and for the analysis of studies conducted in China, it was observed that copper levels were significantly higher in CRC patients compared with healthy controls. Meta-regression analyses revealed no significant influence of the covariates on the pooled estimated effect. The funnel plot showed evidence of publication bias, which was statistically confirmed by Egger’s test (*P* < .001) (Supplementary Figure 1). When evaluating the stability of the results using the trim and fill method with studies imputation of the right side (Supplementary Figure 2), lower copper levels were observed in CRC patients than in controls (SMD = −0.50; 95% CI: −0.98, −0.01). In addition, the cumulative meta-analysis by publication year showed that the pooled result changed over time, specifically from studies published from 2015 onward (Supplementary Figure 3).

**Table 3.** Subgroup analyses for studies of serum/plasma/blood copper and CRC.

Group	Nº of studies	Hedges’s (95% CI)	*P*-value	*I* ^2^ (%)	Meta-regression *P*-value
Continent					
Europe	4	0.59 (0.04, 1.15)	.036	88.41; *P* = .00	.347
Asia	12	−0.18 (−0.81, 0.45)	.568	97.13; *P* = .00	
South America	1	0.69 (0.21, 1.17)	.005	—	
Europe-Asia	2	2.49 (−6.99, 11.97)	.607	99.35; p = 0.00	
Copper determination method					
ICP-MS	3	0.88 (0.64, 1.12)	.000	17.72; *P* = .30	.606
AAS^a^	10	−0.25 (−1.31, 0.80)	.640	98.14; *P* = .00	
Non-standard methods^b^	6	0.21 (−0.17, 0.59)	.277	91.65; *P* = .00	
Country					
China	3	0.75 (0.53, 0.98)	.000	0.00; *P* = .81	.973
India	2	1.70 (0.67, 2.72)	.001	86.17; *P* = .01	
Pakistan	1	−1.31 (-1.55, −1.06)	.000	—	
Iraq	2	−8.31 (24.97, 8.35)	.328	99.12; *P* = .00	
Turkey	2	2.49 (−6.99, 11.97)	.607	99.35; *P* = .00	
Iran	3	−0.28 (−1.04, 0.48)	.468	91.72; *P* = .00	
Brazil	1	0.69 (0.21, 1.17)	.005	—	
Slovak Republic	1	0.20 (−0.18, 0.58)	.310	—	
Italy	1	1.22 (0.74, 1.70)	.000	—	
Spain	1	1.23 (0.31, 2.15)	.009	—	
Saudi Arabia	1	0.02 (-0.17, 0.22)	.797	—	
Multi-European countries	1	0.09 (0.00, 0.18)	.045	—	
Publication year					
Before 2015	6	0.87 (0.24, 1.50)	.007	91.97; *P* = .00	.063
2016–2023	13	−0.12 (−0.75, 0.51)	.707	97.97; *P* = .00	
NOS scale category					
Low	12	0.26 (−0.56, 1.07)	.535	97.37; *P* = .00	.601
Medium	6	−0.03 (−1.04, 0.97)	.947	98.04; *P* = .00	
High	1	0.09 (0.00, 0.18)	.045	—	
Sample size					
*n* < 100	10	0.24 (−0.88, 1.36)	.676	96.92; *P* = .00	.157
*n* > 100	9	-0.09 (-0.61, 0.43)	0.727	97.69; p = 0.00	

Abbreviations: AAS: atomic absorption spectrometry; ICP-MS, inductively coupled plasma mass spectrometry.

^a^Includes both atomic absorption spectrometry and flame atomic absorption spectrometry methods.

^b^Non-standard methods: ICP-OES: inductively coupled plasma optical emission spectrometry, TXRF: total reflection X-ray fluorescence, I-EC: ion-exchange chromatography, colorimetric and spectrophotometric.

In a sensitivity analysis, omitting one study at a time, we identified that Al-Ansari *et al*.’s study [25] was an outlier. Only when this study is omitted, copper concentration was significantly higher in CRC patients than in controls, maintaining a very high heterogeneity (SMD = 0.50; 95% CI: 0.07, 0.92; *I*^2^ = 97.0%; *P* = .00) (Supplementary Figure 4).

No association was found between copper and CRC risk when the levels were measured in a categorical way (in quartiles, quintiles, or others depending on the study) or when increased by a unit of SD (Supplementary Figure 5).

##### Tissue

Only two studies analysed copper in tissue. The pooled effect estimate showed lower copper levels in CRC patients than in healthy subjects (SMD = −1.69; 95% CI: −3.41, 0.03), observing a high heterogeneity (*I*^2^ = 85.56%; *P* = .00) (Figure 2).

##### Diet

Based on only two studies, the pooled effect result of the meta-analysis indicated that CRC patients have a lower dietary intake of copper than controls (SMD = −0.27; 95% CI: −0.51, −0.03), with no evidence of heterogeneity (*I*^2^ = 0.00%; *P* = .95) (Figure 2).

When dietary copper intake was categorized in quartiles, no association was found between dietary copper intake and CRC risk (Supplementary Figure 6).

#### Copper/zinc ratio

**Figure 3:**
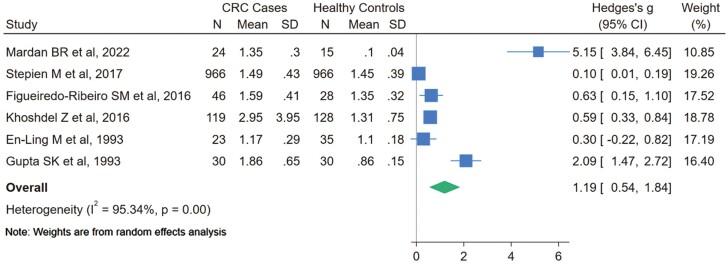
Forest plot of Hedges’s *g* SMD for serum copper/zinc ratio

Six studies analysed the copper/zinc ratio. It was significantly higher in CRC cases than in controls (SMD = 1.19; 95% CI: 0.54, 1.84). However, heterogeneity was very high (*I*^2^ = 95.34%; *P* = .00) (Figure 3). Meta-regression analyses suggested a significant influence of the copper determination method on the estimated pooled effect (*P* = .043). Regarding other methods of copper determination, CRC cases had a significantly higher copper/zinc ratio than controls (*I*^2^ = 0.00%; *P* = .45) (Supplementary Table 5). The funnel plot showed evidence of publication bias, which was statistically confirmed by Egger’s test (*P* < .001) (Supplementary Figure 7).

## Discussion

To our knowledge, the present study is the first meta-analysis to investigate the association between copper measured in two biological matrices (serum/plasma/blood and tissue) and dietary intake and incident CRC risk, along with the effect of copper/zinc ratio on CRC. Specifically, no differences were found between CRC patients and controls with respect to serum/plasma/blood and tissue copper levels and copper/zinc ratio, observing a very high heterogeneity. Regarding dietary copper, CRC patients had a lower intake compared with controls.

With regard to the serum copper levels, our results are consistent with those of a previous meta-analysis by Squitti and colleagues, which found no differences in serum copper levels between colorectal cancer patients and controls [26]. However, some limitations have to be taken into account when interpreting the results of these investigators: (i) they included studies with a cross-sectional design (impossibility of establishing the temporal sequence between exposure and effect and, therefore, causality), with prevalent CRC cases (the disease may have influenced serum copper status). This is why our meta-analysis has a smaller number of studies than Squitti’s work; (ii) some studies meet the inclusion criteria but have not been included. This may be because their search equation was not specific enough, and (iii) they performed a limited analysis of the heterogeneity, not considering potentially relevant sources such as the publication period or the quality of the studies.

Regarding colorectal copper tissue levels, as mentioned above, we found no significant differences between colorectal cancer patients and healthy controls. In the same line, a recent meta-analysis of 13 studies found no difference in copper levels between tumour tissue samples and adjacent healthy tissue in patients with colorectal cancer [50].

Similar results to ours have been reported previously with other types of cancers. Thus, a meta-analysis concluded that breast cancer patients had higher serum copper levels and a higher Cu/Zn ratio than healthy controls [15]. Similarly, two other meta-analyses also described higher serum copper levels and a higher Cu/Zn ratio in lung cancer patients compared with healthy subjects [14,51]. The heterogeneity found in these studies, as in our case, was also high.

The role of copper in the aetiology of CRC is not entirely clear. The mechanism that could explain this could be summarized as follows: as uncontrolled cell growth occurs, metabolic activity increases, requiring higher levels of copper. This copper-dependent cell growth and proliferation mechanism have been termed cuproplasia [16,52,53]. As a result, a cancer patient could present higher copper concentrations than healthy subjects because of their disease. However, it is important to remember that copper can have a detrimental effect on cells. Thus, excess intracellular copper in cancer cells can lead to cell death as a result of the increased oxidative stress. This cell death associated with high levels of copper is known as cuproptosis [52,54]. In this respect, research on the use of copper ionophores, which promote excessive intracellular copper accumulation leading to cancer cell death, appears to be a promising strategy in cancer treatment. However, research on cuproptosis and its relationship to cancer is still at an early stage. Further experimental studies are needed to assess the use of cuproptosis as a therapeutic strategy in the future. Copper chelators have also been shown to be effective as an anticancer strategy by reducing the bioavailability of copper in cancer cells and inhibiting cuproplasia [55]. So, it is difficult to understand the real role of copper on the CRC, copper could be a risk or prevention factor, the cause or consequence, or perhaps there is no association between copper and CCR. It is clear that better methodological studies are necessary [56].

Taking into account the quality of the studies, and concretely how the copper levels are measured, the kind of instrument used to determine what the copper levels are is one key factor. Based on subgroup analyses, our results revealed that serum/plasma/blood copper levels were statistically higher in CRC patients than in controls in those studies that used ICP methods to quantify copper levels and studies conducted in China. This may be due to the analytical advantages of spectrophotometric techniques for determining metals and trace elements over older techniques based on fluorometry, colourimetry, or chromatography. It should also be noted that among spectrophotometric techniques, ICP spectrometry has greater advantages than atomic absorption spectrometry (AAS). Therefore, special attention should be paid to the technique used when comparing copper levels. Inductively coupled plasma mass spectrometry (ICP-MS) has many advantages such as rapid analysis time, low detection limit, clean mass spectra, high spectral resolution, multi-elemental capability, and high sensitivity and accuracy. Additionally, ICP-MS can be hyphenated with high-performance liquid chromatography in order to realize speciation analysis. On the other hand, AAS is relatively inexpensive and easy to use, while still offering high-throughput, quantitative analysis of the metal content of solids or liquids [56].

Our study has some limitations that should be taken into account: (i) very few studies have evaluated copper in tissue or through dietary intake; we only have a large number of studies for copper in serum or plasma. Something similar occurred for the copper/zinc ratio. Similarly, very few studies reported measures of association for CRC risk. This has not allowed us to adequately identify possible sources of heterogeneity and assess potential selection bias; (ii) the development of CRC involves a long period of time, while the determination of copper intake levels over such long periods of time is difficult, so this measure probably does not accurately reflect the level of exposure; and (iii) in most of the included case–control studies, matching was done by age, without considering other CRC risk factors, which could result in biased effect estimates.

Our study has several strengths. First, to our knowledge, this is the first meta-analysis investigating copper levels from different sources, such as serum/plasma/blood, tissue, and dietary intake, in CRC patients compared with healthy controls, joint to copper/zinc ratio. In addition, we evaluated the association between Cu-S and incident CRC risk. Finally, we have included only studies with incident cases, including only those studies in which the determinations were made at the time of diagnosis and before treatment. However, we cannot rule out, due to the scarce information reported by some studies, the possibility of having included some studies where the disease in the cases may have influenced copper toxicokinetic (reverse causality).

## Conclusions

The evidence from this meta-analysis does not suggest that there are differences in copper levels between CRC patients and controls from published studies. However, evidence shows mostly low or moderate quality, and results were quite heterogeneous. New studies with better quality design and better tools to measure the real levels of copper are necessary.

## Supplementary Material

bgaf001_suppl_Supplementary_Figure_S1

bgaf001_suppl_Supplementary_Figure_S2

bgaf001_suppl_Supplementary_Figure_S3

bgaf001_suppl_Supplementary_Figure_S4

bgaf001_suppl_Supplementary_Figure_S5

bgaf001_suppl_Supplementary_Figure_S6

bgaf001_suppl_Supplementary_Figure_S7

bgaf001_suppl_Supplementary_Table_S1

bgaf001_suppl_Supplementary_Table_S2

bgaf001_suppl_Supplementary_Table_S3

bgaf001_suppl_Supplementary_Table_S4

bgaf001_suppl_Supplementary_Table_S5

## Data Availability

The original contributions presented in the study are included in the article/supplementary material, further inquiries can be directed to the corresponding author.
