# Supplementary material for: Copper in colorectal cancer patients: a systematic review and meta-analysis
Source: Carcinogenesis. 2025 Jan 23;46(1):bgaf001. doi: 10.1093/carcin/bgaf001 (PMC11826919; doi:10.1093/carcin/bgaf001)
Supplement: bgaf001_suppl_Supplementary_Figure_S2 [file bgaf001_suppl_supplementary_figure_s2.docx]

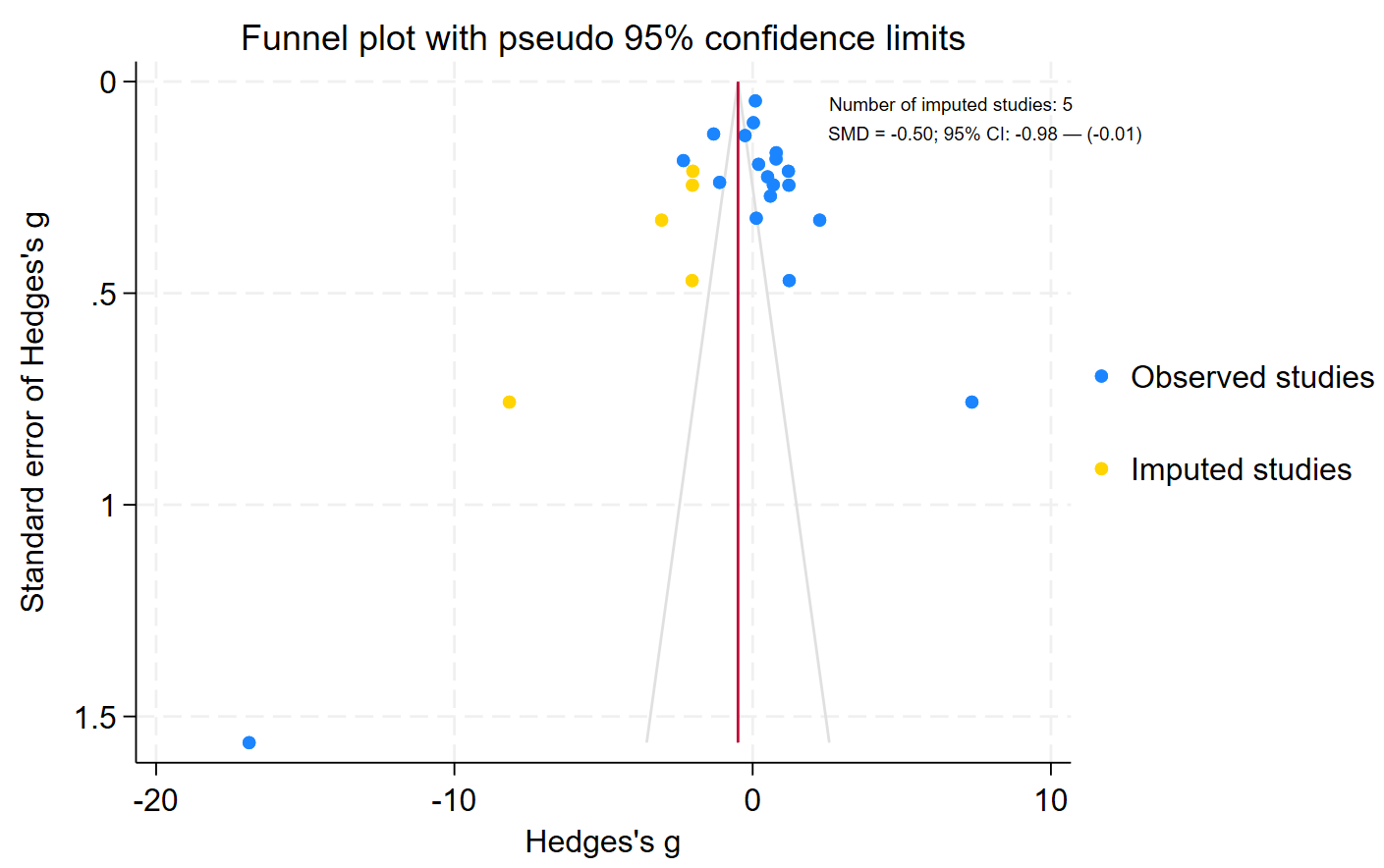


**Supplementary Figure 2.** Trim and fill plot for serum/plasma/blood copper levels showing the imputed missing studies for adjusting funnel plot asymmetry.
