# Supplementary figures and images for: Copper in colorectal cancer patients: a systematic review and meta-analysis
Source: Carcinogenesis. 2025 Jan 23;46(1):bgaf001. doi: 10.1093/carcin/bgaf001 (PMC11826919; doi:10.1093/carcin/bgaf001)

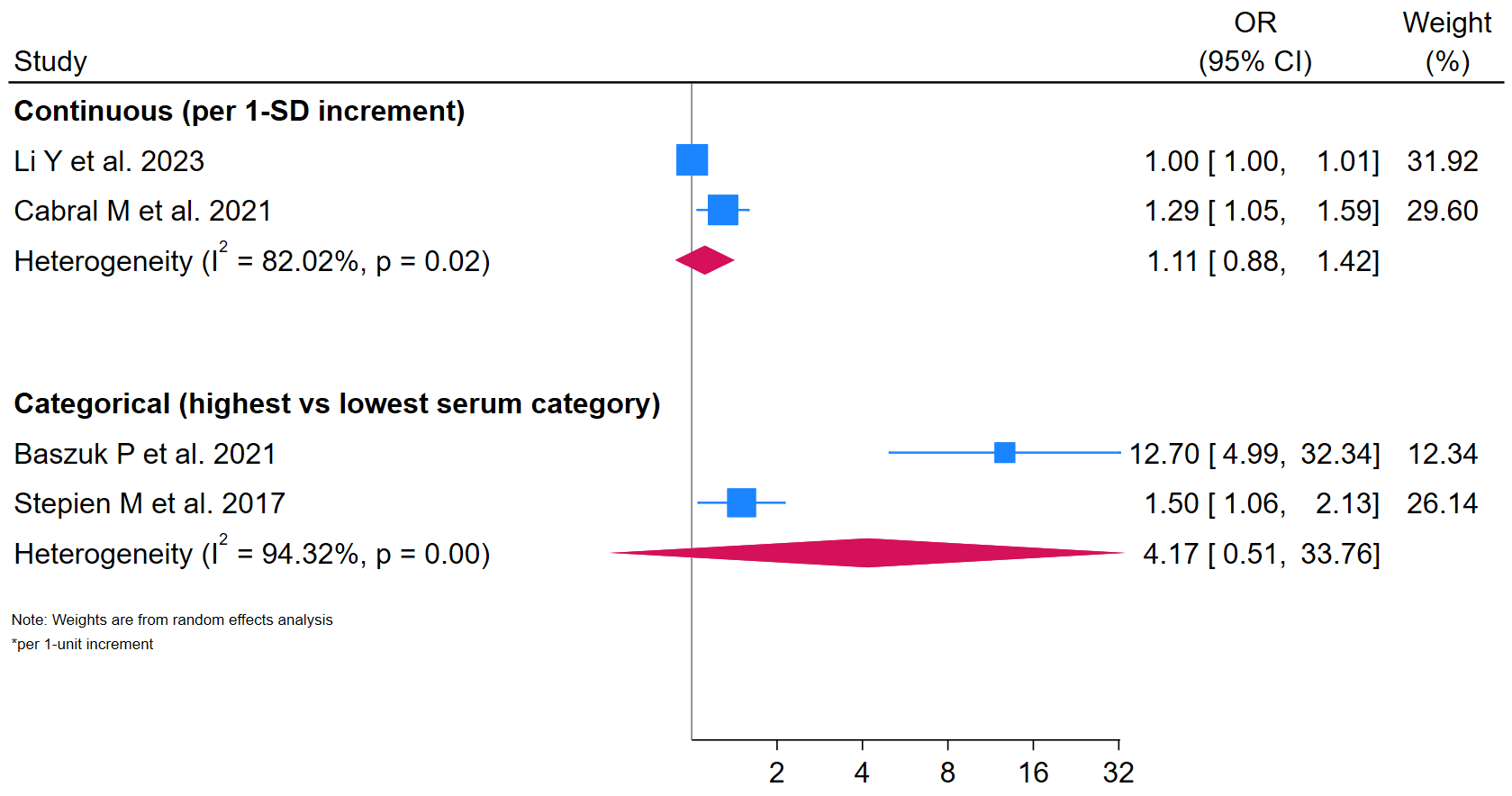


**Supplementary Figure 5**. Forest plot of the association between serum/blood copper and CRC

Supplement: bgaf001_suppl_Supplementary_Figure_S5 [file bgaf001_suppl_supplementary_figure_s5.docx]

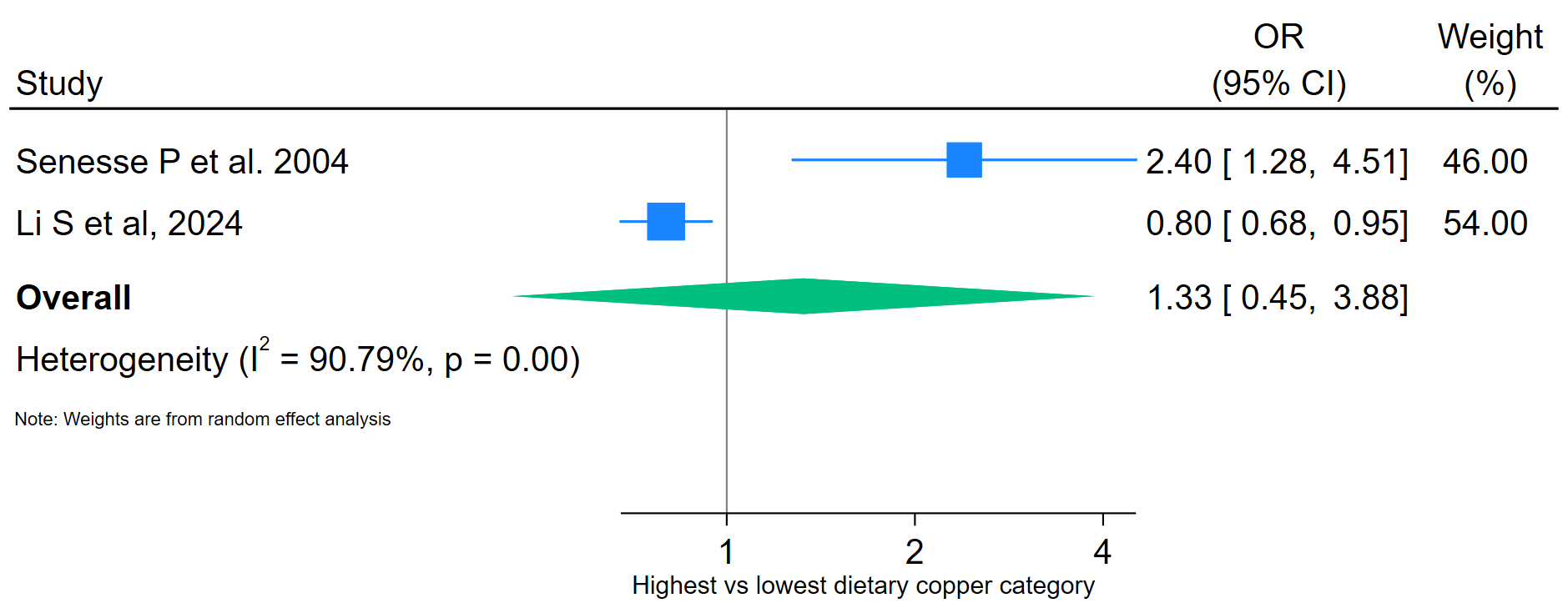


**Supplementary Figure 6**. Forest plot of the association between dietary copper intake and CRC.

Supplement: bgaf001_suppl_Supplementary_Figure_S6 [file bgaf001_suppl_supplementary_figure_s6.docx]
