# Supplementary material for: Copper in colorectal cancer patients: a systematic review and meta-analysis
Source: Carcinogenesis. 2025 Jan 23;46(1):bgaf001. doi: 10.1093/carcin/bgaf001 (PMC11826919; doi:10.1093/carcin/bgaf001)
Supplement: bgaf001_suppl_Supplementary_Table_S1 [file bgaf001_suppl_supplementary_table_s1.docx]

**Supplementary Table 1**. PECOS strategy

| **Variable** | **Definition** |
| --- | --- |
| (P) Participants | Men and women older than 18 years old |
| (E) Exposure | Copper measured in two biological matrices (serum/plasma/blood and tissue) and dietary intake |
| (C) Comparator | Participants without exposure or with low copper exposure |
| (O) Outcome | Colorectal cancer |
| (S) Study | Case-control and cohort study |
