# Supplementary material for: Copper in colorectal cancer patients: a systematic review and meta-analysis
Source: Carcinogenesis. 2025 Jan 23;46(1):bgaf001. doi: 10.1093/carcin/bgaf001 (PMC11826919; doi:10.1093/carcin/bgaf001)
Supplement: bgaf001_suppl_Supplementary_Table_S2 [file bgaf001_suppl_supplementary_table_s2.docx]

**Supplementary Table 2**. Search strategy.

|  | **PubMed** | **Embase** | **Scopus** |
| --- | --- | --- | --- |
| Date | 07/11/2023 | 07/11/2023 | 07/11/2023 |
| #1 | (copper[MeSH Terms] OR copper[Title/Abstract] OR copper level*[Title/Abstract] OR copper concentration*[Title/Abstract] OR trace elements[MeSH Terms] OR trace element*[Title/Abstract] OR heavy metal*[Title/Abstract] OR mineral intake*[Title/Abstract] OR nutrients intake*[Title/Abstract]) | (“copper”:ab,ti OR “copper level*”:ab,ti OR “copper concentration*”:ab,ti OR “trace element*”:ab,ti OR “heavy metal*”:ab,ti OR “mineral intake*”:ab,ti OR “nutrients intake*”:ab,ti) | (TITLE-ABS (("copper") OR ( "copper level*" ) OR ( "copper concentration*" ) OR ( "trace element*" ) OR ( "heavy metal*" ) OR ( "mineral intake*" ) OR ( "nutrients intake*" ))) |
| #2 | (colon cancer[Title/Abstract] OR colon tumor[Title/Abstract] OR colon carcinoma[Title/Abstract] OR colon malignancy[Title/Abstract] OR colorectal cancer[Title/Abstract] OR colorectal tumor[Title/Abstract] OR colorectal carcinoma[Title/Abstract] OR colorectal malignancy[Title/Abstract] OR colorectal neoplasms[MeSH Terms] OR colonic neoplasms[MeSH Terms]) | (“colon cancer”:ab,ti OR “colon tumor”:ab,ti OR “colon carcinoma”:ab,ti OR “colon malignancy”:ab,ti OR “colorectal cancer”:ab,ti OR “colorectal tumor”:ab,ti OR “colorectal carcinoma”:ab,ti OR “colorectal malignancy”:ab,ti OR “colorectal neoplasms”:ab,ti OR “colonic neoplasms”:ab,ti) | (TITLE-ABS (( "colon cancer" ) OR ( "colon tumor" ) OR ( "colon carcinoma" ) OR ( "colon malignancy" ) OR ( "colorectal cancer" ) OR ( "colorectal tumor" ) OR ( "colorectal carcinoma" ) OR ( "colorectal malignancy" ) OR ( "colorectal neoplasm" ) OR ( "colonic neoplasm"))) |
|  | (#1 AND #2) | | |

|  | **Web Of Science** |  |  |
| --- | --- | --- | --- |
| Date | 12/11/2024 |  |  |
| #1 | (TI="copper" OR AB="copper" OR TI="copper level*" OR AB="copper level*" OR TI="copper concentration*" OR AB=" copper concentration*" OR TI="trace element*" OR AB="trace element*" OR TI="heavy metal*" OR AB=" heavy metal*" OR TI="mineral intake*" OR AB=" mineral intake*" OR TI="nutrients intake*" OR AB="nutrients intake*") |  |  |
| #2 | (TI="colon cancer" OR AB="colon cancer" OR TI="colon tumor" OR AB=" colon tumor" OR TI="colon carcinoma" OR AB="colon carcinoma" OR TI="colon malignancy" OR AB="colon malignancy" OR TI="colorectal cancer" OR AB="colorectal cancer" OR TI="colorectal tumor" OR AB="colorectal tumor" OR TI="colorectal carcinoma" OR AB="colorectal carcinoma" OR TI="colorectal malignancy" OR AB="colorectal malignancy" OR TI="colorectal neoplasm" OR AB="colorectal neoplasm" OR TI="colonic neoplasm" OR AB="colonic neoplasm") |  |  |
|  | (#1 AND #2) | | |
