# Supplementary material for: Copper in colorectal cancer patients: a systematic review and meta-analysis
Source: Carcinogenesis. 2025 Jan 23;46(1):bgaf001. doi: 10.1093/carcin/bgaf001 (PMC11826919; doi:10.1093/carcin/bgaf001)
Supplement: bgaf001_suppl_Supplementary_Table_S3 [file bgaf001_suppl_supplementary_table_s3.docx]

**Supplementary Table 3**. List of excluded studies by full-text screening

| **Exclusion reason** | **First author (year)** | **Title** |
| --- | --- | --- |
| Not suitable design (prevalent CRC cases) | Zowczak-Drabarczyk M, 2024 | Trace Element Levels in Blood Serum and Colon Tissue in Colorectal Cancer |
|  | Zhang C, 2022 | Serum Copper and Zinc Levels and Colorectal Cancer in Adults: Findings from the National Health and Nutrition Examination 2011–2016. |
|  | Ay A, 2021 | Investigation of the relationship between GSTM1 gene variations and serum trace elements, plasma malondialdehyde levels in patients with colorectal cancer. |
|  | Milde D, 2007 | Evaluation of Colon Cancer Elements Contents in Serum Using Statistical Methods. |
|  | Milde D, 2005 | Trace Element Levels in Blood Serum and Colon Tissue in Colorectal Cancer. |
|  | Nayak S, 2003 | Copper and ceruloplasmin status in serum of prostate and colon cancer patients. |
|  | Milde D, 2001 | Serum levels of selenium, manganese, copper, and iron in colorectal cancer patients. |
|  | Narang A, 1990 | Copper and zinc levels in the serum of patients with esophageal, gastric and colorectal carcinoma. |
| Not suitable study design (samples in healthy and cancer tissue from the same patient) | Gregoriadis G, 1983 | A comparative study of trace elements in normal and cancerous colorectal tissues. |
| Not exposure of interest (no copper measurement) | Gholamalizadeh M, 2023 | The association between colorectal cancer and index of nutritional quality (INQ); a case- control study. |
|  | Vahid F, 2022 | The association between dietary total antioxidant capacity and quality of nutrients with odds of colorectal cancer: A hospital-based case–control study. |
|  | Augustyniak M, 2022 | Calcium intake may explain the reduction of colorectal cancer odds by dietary selenium - a case-control study in Poland. |
|  | Papadimitriou N, 2022 | A Prospective Diet-Wide Association Study for Risk of Colorectal Cancer in EPIC |
|  | Alves Ribeiro R, 2022 | Risk of Colorectal Cancer in a Brazilian Population is Differentially Associated with the Intake of Processed Meat and Vitamin E. |
|  | Tsilidis K, 2021 | Genetically predicted circulating concentrations of micronutrients and risk of colorectal cancer among individuals of European descent: A Mendelian randomization study. |
|  | Swaminath S, 2019 | Combined mineral intakes and risk of colorectal cancer in postmenopausal women. Metals distribution in colorectal biopsies: New insight on the elemental fingerprint of tumour tissue. |
|  | Rinaldi L, 2015 | Macro- and micronutrients consumption and the risk for colorectal cancer among Jordanians. Does dietary calcium interact with dietary fiber against colorectal cancer? A case-control study in Central Europe. |
|  | Tayyem R, 2015 | Dietary intake of calcium, fiber and other micronutrients in relation to colorectal cancer risk: Results from the Shanghai Women’s Health Study. |
|  | Galas A, 2013 | Folate, Vitamin B6, Vitamin B12, and Vitamin B2 Intake, Genetic Polymorphisms of Related Enzymes, and Risk of Colorectal Cancer in a Hospital-Based Case-Control Study in Japan. |
|  | Shin A, 2006 | Diet and risk of colon cancer in a large prospective study of older women: An analysis stratified on family history (Iowa, United States). |
|  | Otani T, 2005 | Dietary fiber and colorectal cancer risk. |
|  | Sellers T, 1998 | Calcium, Vitamin D, and the Occurrence of Colorectal Cancer Among Women. |
|  | Marchand L, 1997 | The association between colorectal cancer and index of nutritional quality (INQ); a case- control study. |
|  | Martínez M, 1996 | The association between dietary total antioxidant capacity and quality of nutrients with odds of colorectal cancer: A hospital-based case–control study. |
| Not outcome (other cancer) | Ma E, 1992 | Ion-exchange chromatography (IC) in the determination of serum transition metals and its clinical application. |
|  | Danielsen A, 1970 | A study of some selected trace elements in normal and cancerous tissue by neutron activation analysis. |
| Not in English | Klimczak A, 2009 | Activity of selected antioxidants enzymes examination in people with colorectal cancer. Nutritional and antioxidant status of colorectal tumor patients. |
|  | Regöly-Mérei A, 2007 | Study on the association and significance between trace elements and rectal cancer. Precancerous conditions and carcinomas of the stomach and colorectum--blood levels of selected micronutrients. |
|  | Han C, 1999 | Relationship between colorectal cancer and ten inorganic elements. |
|  | Beno I, 1997 | Level of copper in plasma and tissue of patients with esophageal and large bowel cancer. |
|  | Yang G, 1993 | Activity of selected antioxidants enzymes examination in people with colorectal cancer. Nutritional and antioxidant status of colorectal tumor patients. |
|  | Witkowski K, 1993 | Study on the association and significance between trace elements and rectal cancer. Precancerous conditions and carcinomas of the stomach and colorectum--blood levels of selected micronutrients. |
| No full-text access | Rutkowski M, 2010 | Evaluation of selected microelements concentrations in plasma of colorectal carcinoma patients. |
|  | Feng J, 2006 | Correlation between oxidative stress and trace elements in blood of patients with cancer. Clinical evaluation of serum trace elements in colorectal cancer. |
|  | Wang H, 1995 | Correlation between oxidative stress and trace elements in blood of patients with cancer. Clinical evaluation of serum trace elements in colorectal cancer. |
| Other reasons (congress abstract) | Sandra P, 2023 | Role of trace elements in early detection of cancer. |
|  | Luu H, 2022 | Dietary potassium and risk of colorectal cancer: Findings from the Singapore Chinese Cohort Study. |
|  | Keskin H, 2018 | Risk factors of colorectal cancer in Linxian, China: A nutrition intervention trial with 30 years follow-up. |
|  | Zowczak-Drabarczyk M, 2016 | The clinical utility of serum Se, Zn, Cu and plasma TAS determination in colorectal cancer and adenoma in Polish population. |
|  | Ay A, 2015 | Investigation of GSTM1 polymorphism genotypes and serum trace elements in colorectal cancer. |
|  | ALfaris, 2013 | Trace elements of colon cancer-a case control study. |
| Other reasons (Same sample as one of the included studies) | Beňo I, 2001 | Blood levels of natural antioxidants in gastric and colorectal precancerous lesions and cancers in Slovakia. |
