# Supplementary material for: Copper in colorectal cancer patients: a systematic review and meta-analysis
Source: Carcinogenesis. 2025 Jan 23;46(1):bgaf001. doi: 10.1093/carcin/bgaf001 (PMC11826919; doi:10.1093/carcin/bgaf001)
Supplement: bgaf001_suppl_Supplementary_Table_S4 [file bgaf001_suppl_supplementary_table_s4.docx]

**Supplementary Table 4**. Quality assessment of the studies included in the systematic review and meta-analysis

| **Case-control studies** | | | | | | | | | | | | |
| --- | --- | --- | --- | --- | --- | --- | --- | --- | --- | --- | --- | --- |
|  | | **SELECTION** | | | | **COMPARABILITY** | | **EXPOSURE** | | |  | |
| **First Author** | **Year** | **Definition of cases** | **Representativeness of cases** | **Selection of controls** | **Definition of controls** | **Important Factor** | **Additional Factor** | **Ascertainment** | **Same methods for subjects** | **Non-response rate** | **NOS score** | |
| **Li Y** | **2023** | * | * | - | * | * | - | * | * | * | 7 | Moderate |
| **Saxena A** | **2023** | * | * | - | * | * | - | * | * | * | 7 | Moderate |
| **Mahmood MHR** | **2022** | * | - | - | * | - | - | * | * | - | 4 | Low |
| **Mardan BR** | **2022** | - | - | - | * | - | - | * | * | * | 4 | Low |
| **Türkdogan MK** | **2022** | * | - | - | * | - | - | * | * | * | 5 | Low |
| **Baszuk P** | **2021** | * | * | - | * | * | - | * | * | * | 7 | Moderate |
| **Nozadi F** | **2021** | * | * | - | * | * | - | * | * | * | 7 | Moderate |
| **Al-ansari RF** | **2020** | * | - | - | * | - | - | * | * | * | 5 | Low |
| **Natajomrani RA** | **2020** | * | - | - | * | * | - | * | * | * | 6 | Moderate |
| **Nawi AM** | **2020** | * | - | - | * | - | - | * | * | * | 5 | Low |
| **Ranjbary AG** | **2020** | * | - | - | * | - | - | * | * | * | 5 | Low |
| **Wang H** | **2020** | - | - | - | - | - | - | * | * | * | 3 | Low |
| **Stepien M** | **2017** | * | * | * | * | * | * | * | * | * | 9 | High |
| **Figueiredo-Ribeiro SM** | **2016** | * | - | - | * | - | - | * | * | * | 5 | Low |
| **Khosdel Z** | **2016** | * | - | - | * | - | - | * | * | * | 5 | Low |
| **Kucukhuseyin O** | **2015** | * | - | - | * | * | - | * | * | * | 6 | Moderate |
| **Al Faris NA** | **2011** | * | - | - | * | * | - | * | * | * | 6 | Moderate |
| **Senesse P** | **2004** | * | * | * | * | * | - | * | * | - | 7 | Moderate |
| **Magálová T** | **1999** | * | - | - | * | - | - | * | * | * | 5 | Low |
| **Stefanati A** | **1995** | * | * | - | * | - | - | * | * | * | 6 | Moderate |
| **En-ling M** | **1993** | * | - | - | - | - | - | * | * | * | 4 | Low |
| **Gupta SK** | **1993** | * | - | - | * | - | - | * | * | * | 5 | Low |
| **Martín-Mateo MC** | **1988** | * | - | - | * | - | - | * | * | - | 4 | Low |

| **Cohort studies** | | | | | | | | | | | | |
| --- | --- | --- | --- | --- | --- | --- | --- | --- | --- | --- | --- | --- |
|  | | **SELECTION** | | | | **COMPARABILITY** | | **OUTCOME** | | |  |  |
| **First Author** | **Year** | **Representativeness of exposed cohort** | **Representativeness of unexposed cohort** | **Ascertainment of exposure** | **Outcome was not present at start** | **Important Factor** | **Additional Factor** | **Assessment** | **Exposure follow-up long enough for outcomes to occur** | **Adequacy of follow-up** | **Total score** | **NOS categorical scale** |
| **Jin D** | **2023** | * | * | - | * | * | * | * | * | * | 8 | High |
| **Cabral M** | **2021** | * | * | * | - | * | * | * | - | * | 7 | Moderate |
| **Li S** | **2024** | - | * | - | * | * | * | - | * | * | 6 | Moderate |
