# Supplementary material for: Copper in colorectal cancer patients: a systematic review and meta-analysis
Source: Carcinogenesis. 2025 Jan 23;46(1):bgaf001. doi: 10.1093/carcin/bgaf001 (PMC11826919; doi:10.1093/carcin/bgaf001)
Supplement: bgaf001_suppl_Supplementary_Table_S5 [file bgaf001_suppl_supplementary_table_s5.docx]

**Supplementary Table 5.** Subgroup analyses for Copper/Zinc ratio and CRC

| **Group** | **Nº of studies** | **Hedges’s (95% CI)** | **P value** | **I^2^ (%)** | **Meta-regression P value** | |
| --- | --- | --- | --- | --- | --- | --- |
| **Continent** | | | | | | |
| Europe | 1 | 0.10 (0.01, 0.19) | 0.032 | — | | 0.664 |
| Asia | 4 | 1.86 (0.60, 3.13) | 0.004 | 95.40; p = 0.00 | |  |
| South America | 1 | 0.63 (0.15, 1.10) | 0.010 | — | |  |
| **Copper determination method** | | | | | | |
| AAS | 4 | 1.93 (0.74, 3.11) | 0.001 | 95.15; p = 0.00 | | 0.043 |
| Other | 2 | 0.10 (0.02, 0.19) | 0.021 | 0.00; p = 0.45 | |  |
| **NOS scale category** | | | | | | |
| Low | 5 | 1.55 (0.6, 2.47) | 0.001 | 93.93; p = 0.00 | | 0.188 |
| High | 1 | 0.10 (0.0, 0.19) | 0.032 | — | |  |

AAS: atomic absorption spectrometry.
